# Supplementary material for: Resolving the full spectrum of human genome variation using Linked-Reads
Source: Genome Res. 2019 Apr;29(4):635–45. doi: 10.1101/gr.234443.118 (PMC6442396; doi:10.1101/gr.234443.118)
Supplement: Supplemental Material [file supp_gr.234443.118_Supplemental_longranger-2.2.zip › longranger-2.2.tar.bz2!/lr2.2-public/lib/stan/cmdstan/stan/doc/api/html/likely_8hpp.html]

Stan: src/stan/meta/likely.hpp File Reference


|  |  |
| --- | --- |
|  | Stan  2.5.0  probability, sampling & optimization |


- Main Page
- Related Pages
- Namespaces
- Classes
- Files

- File List
- File Members

All Classes Namespaces Files Functions Variables Typedefs Enumerations Enumerator Friends Macros Pages

- src
- stan
- meta

Macros

likely.hpp File Reference

Go to the source code of this file.

|  |  |
| --- | --- |
| Macros | |
| #define | likely(x)   (x) |
|  | |
| #define | unlikely(x)   (x) |
|  | |

## Macro Definition Documentation

|  |  |  |  |  |  |
| --- | --- | --- | --- | --- | --- |
| #define likely | ( |  | x | ) | (x) |

Definition at line 8 of file likely.hpp.

|  |  |  |  |  |  |
| --- | --- | --- | --- | --- | --- |
| #define unlikely | ( |  | x | ) | (x) |

Definition at line 9 of file likely.hpp.


---

[ Stan Home Page ]

*© 2011–2014,
Stan Development Team.*
